# Analysis of 3-MCPD and 1,3-DCP occurrence in mayonnaise: A probabilistic risk assessment of dietary exposure for Iranians

**DOI:** 10.1016/j.toxrep.2024.101725

**Published:** 2024-08-31

**Authors:** Seyedeh Faezeh Taghizadeh, Christina Tsitsimpikou, Aristidis Tsatsakis, Hadi Haghparast, Ghazaleh Tabriznia Tabrizi, Mahin Velayati, Gholamreza Karimi, Ramin Rezaee

**Affiliations:** a Applied Biomedical Research Center, Mashhad University of Medical Sciences, Mashhad, Iran; b General Chemical State Laboratory of Greece, Athens 11512, Greece; c Laboratory of Toxicology, Medical School, University of Crete, Heraklion, Crete, Greece; d Student Research Committee, Mashhad University of Medical Sciences, Mashhad, Iran; e Department of Pharmacodynamics and Toxicology, School of Pharmacy, Mashhad University of Medical Sciences, Mashhad, Iran

**Keywords:** Chloropropanols, Chronic oral exposure, High fat mayonnaise, Low fat mayonnaise, Regular daily intake

## Abstract

Mayonnaise is a commonly used oil-in-water emulsion food product. Due to their toxicological properties/genotoxicity and carcinogenicity, chloropropanols’ oral exposure has raised concerns over the past decade. The present study reports the occurrence level of free forms of 3-chloropropane-1,2-diol (3-MCPD) and 1,3-dichloro-2-propanol (1,3-DCP) in mayonnaise samples and the risk of oral exposure to these chemicals through consumption of the analyzed samples. Mayonnaise (low- and high-fat, from 6 brands, totally 120 samples) were analyzed for 1,3-DCP and 3-MCPD by gas chromatography-mass spectrometry. The mean level of chemicals was higher in the high-fat samples, with no significant difference among the brands. Generally, 1,3-DCP level was significantly lower in both high-fat and low-fat samples compared to 3-MCPD. Hazard Index (HI) values calculated for oral exposure to 3-MCPD for Iranian adults using probabilistic methods, were less than 1.0, reflecting no major risk*.* In the Margin of Exposure scenario, low- and high-fat mayonnaise samples were of *de minimis* health concern at the 50th, 80th, and 95th centiles. Nevertheless, in order to safeguard consumer interests, it is imperative to implement online real-time methodologies for monitoring reactions that result in generation of thermal process contaminants such as 3-MCPD and 1,3-DCP, and to innovate novel technologies to minimize the occurrence of such chemicals while preserving both safety and sensory attributes.

## Highlights

• The levels of 3-MCPD and 1,3-DCP were assessed in mayonnaise from Iran.
• Hazard Indices were <1, indicating no risk for consumers’ health.
• 1,3-DCP level was significantly lower in high and low-fat samples compared to 3-MCPD.

## Graphical Abstract

**Figure:**
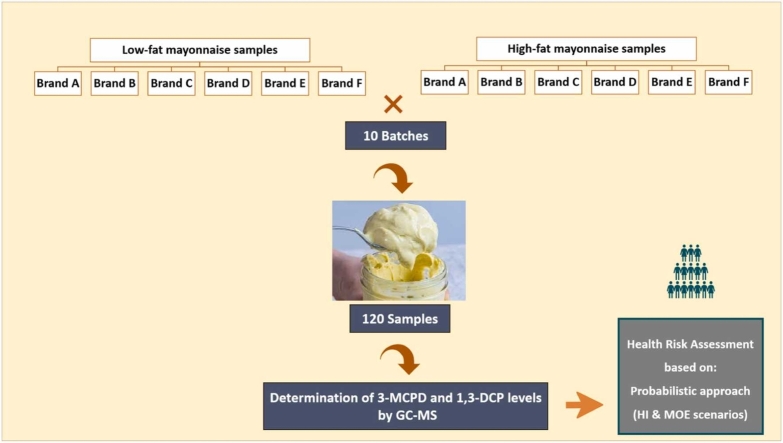

## 1 Introduction

From the chloropropanols family, 3-chloropropane-1,2-diol (3-MCPD) and 1,3-dichloro-2-propanol (1,3-DCP) have been singled out as contaminants in oil-based and heat-processed foods. These particular substances were initially identified in protein hydrolysates. Typically, 1,3-DCP content is lower compared to 3-MCPD, with its formation occurring naturally under conditions where the 3-MCPD concentration is elevated [21]. It has been elucidated that monochloropanol amount generated during food processing is typically 100–10,000 times higher than that of dichloropanol. Similarly, the concentration of 3-MCPD is commonly several to tenfold greater than that of 2-chloro-1,3-propanediol. As a result, the assessment of 3-MCPD levels in food serves as a proxy for the production of chloropropanols in food processing [16]. During digestion, 3-MCPD is released from its esterified form and becomes the free form of 3-MCPD. Various factors/conditions including the level of acylation, chlorination, and the ratio of isomers in the chlorinated compounds were reported to affect 3-MCPD formation [29]. It was suggested that salts of hydrogen chloride and free fatty acids (FFA) may be created during the deodorization process when fatty acids are in balance with chloride ions like potassium chloride, sodium chloride, and tetraethylammonium chloride. When chloride ions are present, there is potential for a two-way transformation between 3-MCPD and glycidol or their esterified forms [29]. The occurrence of 1,3-DCP in manufacturing and processing of food products has been reported [19]. Also, there is considerable amount of evidence on the occurrence of chloropropanols in vegetable oils [2], [34], [9]. Various methods have been implemented in the food industry to reduce the presence of harmful chemicals, such as adjusting deodorization temperature, incorporating chelating agents, modifying processing conditions, and more. Despite these efforts, these methods typically do not yield desirable outcomes [25]. Physical refining methods have shown effectiveness in reducing glycidyl esters but have been less successful in addressing 3-MCPD [25]. From a toxicological point of view, 3-MCPD can produce untoward effects in the kidney and reproductive organs even at low concentrations [23]. Benchmark dose (BMD) analysis using model averaging resulted in a benchmark dose lower confidence limit 10 % (BMDL10) of 0.20 mg/kg bw per day in male rats, which was selected as the new Reference Point for renal effects [11]. Also, both compounds are considered possible (Group 2B) carcinogens [18]. Furthermore, the European Food Safety Authority (EFSA), the Joint FAO/WHO Expert Committee on Food Additives (JECFA), and Food Standards Australia New Zealand (FSANZ) established a tolerable daily intake (TDI) of 2 μg/kg body weight (BW) per day for chloropropanols based on a long-term study done in rats [11], [13], [22]. On the other hand, JECFA evaluated the currently available studies of 1,3- DCP toxicity and classified 1,3- DCP as genotoxic *in vitro*; thus, it was concluded that the establishment of a TDI for 1,3-DCP was inappropriate because it is thought to directly damage genetic material and therefore, it is not possible to establish a safe level of consumption (Available at: https://www.reading.ac.uk/foodlaw/news/eu-01125.htm). In 2006, the Committee established a BMDL10 of 3.3 mg/kg BW/day for 1,3-DCP due to concerns about its carcinogenicity and the inability to rule out a genotoxic potential for this chloropropanol [6]. However, male and female rats have shown clear carcinogenic effects when exposed to doses of 1,3-DCP ≥6.3 mg/kg/day. It was stated that high consumers have dietary exposure to 1,3-DCP that is approximately 200,000 times lower than the levels that led to tumor development in animal studies, indicating a very low health and safety risk for consumers [13], [4]. Several studies have shown that the Margin of Exposure (MOE) values for dietary exposure to 1,3-DCP are very high, exceeding 10,000. The high MOE values for 1,3-DCP suggest that its exposure is not a major concern for human health. Together, the health risks associated with the carcinogenicity of 1,3-DCP are considered to be low [38], [4], [42].

Mayonnaise is a popular food item globally due to its pleasing taste. The ingredients used in commercial mayonnaise can vary, but typically include vegetable oils, acidulants, and egg yolks. The oils are the basic components of mayonnaise due to their physicochemical aspect and sensory properties [10]. To our knowledge, there is no report of the occurrence of 3-MCPD and 1,3-DCP (i.e. the free forms of 3-MCPD and 1,3-DCP) in mayonnaise samples from Iran. Thus, this study determined the level of these chemicals in low- and high-fat mayonnaise products available in the Iranian market and assessed the risk of oral exposure to them for Iranian population under a probabilistic scenario using Monte Carlo simulation (MCS).

## 2 Materials and methods

### 2.1 Chemicals

The chemicals 1,3-DCP and 3-MCPD (98 % purity), trimethylsilyl trifluoromethanesulfonate (TMSOTf, 99 % purity), hexamethyldisilazane (HMDS, 99 % purity), and 1,5-pentanediol, Ethyl acetate (EtOAc) and dichloromethane (98 % purity) were purchased from Sigma–Aldrich (Steinheim, Germany).

### 2.2 Sampling

**Fig. 1:**
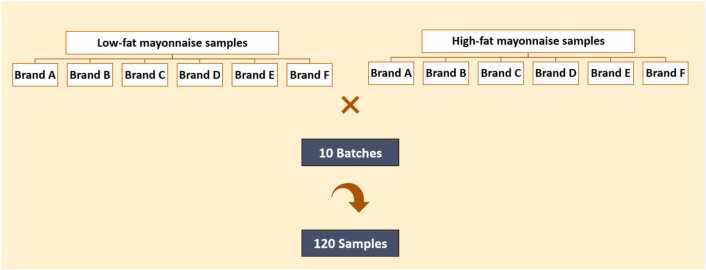
Schematic presentation of sample collection.

From six main mayonnaise-producing brands (6 brands × 10 batches from each brand × 2 types including low-fat and high-fat), as presented in Fig. 1, a total of 120 mayonnaise samples collected from Iran market were analyzed. The collected mayonnaise products were kept at 4 °C until analyses.

### 2.3 Sample preparation and extraction

We employed trimethylsilyl (TMS) derivatization of 1,3-DCP and 3-MCPD using TMSOTf and HMDS, to determine their levels in low- and high-fat mayonnaise products. First, the samples were blended for better homogenization, then, 2.0 g of each sample was placed in a 50.0-mL centrifuge tube containing 8.0 g aluminum oxide. For determination of recovery percentage, spiking with two levels of each of the chloropropanols (0.5 and 1.0 mg/kg) was done. After gentle mixing, samples were placed in a glass chromatography column and a sintered disk without porosity. Prior to adding the sample, the column was loaded with anhydrous sodium sulfate (1.0 g) and cotton (1.0 g) soaked in dichloromethane. Next, the glass chromatography column was eluted with dichloromethane (8.0 mL/min) and the eluent was concentrated and almost dried by purified N_2_ gas. Immediately afterward, the extract was treated with EtOAc (1.0 mL). For derivatization, TMSOTf and HMDS (10.0 and 50.0 µL, respectively) were added to the EtOAc mixture in a vial, and shaken for 45 sec. The derivatization procedure length was 10.0 min and it was done at room temperature (25 °C). To terminate the derivatization, we added water (1.0 mL) and the vial was shaken for 30 sec. Finally, the organic layer was collected in a GC vial, sodium sulfate (a small amount) was added to the same vial to remove any moisture and the samples was analyzed by GC-MS [39].

### 2.4 GC-MS analysis

A quadrupole GC-MS instrument from Agilent, Santa Clara, USA, that was equipped with a DB-5MS capillary column (length 30.0 m, diameter 0.23 mm and film thickness 0.25 mm) was employed in the current work. Here, 1.0 L of the derivatized sample was injected into the GC oven (the inlet temperature 270 °C, which was held at 60 °C for 2.0 min, increased at a rate of 5 °C/min to120 °C, increased to 300 °C at a rate of 30 °C/min and finally, kept at 300 °C for 8.0 min. Purified nitrogen (flow rate 1.0 mL/min) was employed as the carrier gas. The entire temperature program length was about 12.0 min. In addition, characteristic ions with *m/z* values of 116, 119 and 147 for 3-MCPD-TMS and 93, 151 and 154 for 1,3-DCP-TMS were considered for qualification. For quantification the derivatized chemicals, the characteristic ions were considered in single ion monitoring mode [39].

### 2.5 Validation of the current method

In order to determine limit of detection (LOD) and limit of quantitation (LOQ), signal-to-noise ratios of 3.0 and 10.0, respectively were considered. By employing spiked calibration curves plotted for triplicated samples spiked with 0.5 and 1.0 mg/kg of either 3-MCPD or 1,3-DCP, the recovery of the present method was determined [32], [43].

### 2.6 Assessment of risk of oral exposure to 3-MCPD or 1,3-DCP

#### 2.6.1 Hazard index scenario

The estimated daily intake (EDI, mg/kg BW) of the chemicals through mayonnaise intake by Iranians was calculated (Eq. 1) [37]:

(1) $$\;\mathrm{EDIi}=\frac{F\;\times C}{\mathrm{BW}}\;$$

Here, C is 1,3-DCP and 3-MCPD concentrations (mg per kg mayonnaise) and F is the mayonnaise daily intake reported for Iranians (0.0082 kg/day) [15]. For the adult Iranian population, the average BW of 70.0 kg was considered [35]. By dividing EDI by tolerable daily intake (TDI, 0.002 mg/kg body weight/day) [11] the Target Hazard Quotient (THQ) was calculated (Eq. 2). Next, based on the calculated THQ, the hazard index (HI) was determined (Eq. 3) [33]. While a THQ below 1 reflects no health risk following certain exposure, a value >1 highlights potential health risks [12], [31].

(2) $$\mathrm{THQ}=\frac{\mathrm{EDI}}{\mathrm{TDI}\;}\;$$

(3) $$\mathrm{HI}=\sum_{i=1}^{n}{\mathrm{THQ}}_{i\;}$$

#### 2.6.2 Margin of exposure (MOE) scenario

Under this scenario, the risk of oral exposure to 3-MCPD was calculated using a benchmark dose (BMDL_10_) concept. Based on renal effects of 3-MCPD in male rats, a BMDL_10_ of 0.20 mg/kg BW per day has been reported by the EFSA; MOE was calculated as follows (Eq. 4) [11], [14] (https://efsa.onlinelibrary.wiley.com/doi/pdf/10.2903/j.efsa.2018.5083).

(4) $$\mathrm{MOE}=\frac{\mathrm{BMDL}10}{\mathrm{EDI}\;}\;$$

MOE ≤10,000: Reflects an “of concern” health risk

MOE ˃10,000: Reflects a “*de minimis”* health risk

### 2.7 Probabilistic risk assessment

Unlike traditional risk assessment methodologies where only the deterministic data was considered, probabilistic risk assessment methodologies utilize different risk characterization approaches. Probabilistic are less conservative and more realistic compared to the deterministic approached, as they estimate the distribution of potential risk for an individual, or the range of probable risk across a population from least to most at risk. In the present study, MCS was used to assess uncertainties and their impact on the estimated risk. This probabilistic modeling allows to consider a distribution of exposure or risk [36]. JMP 9 software (Campus Drive, Cary, NC 27513) was used to fit the third parameters from the preliminary results with a suitable distribution before the MCS was used to assess the goodness of fit. In this study, the MCS technique was run through 10,000 iterations and used these individual exposure variable distributions as input parameters to determine the probability functions for daily human exposures [30].

### 2.8 Statistical analysis

Mean levels of 1,3-DCP and 3-MCPD were statistically analyzed using JMP 9 software. By post-test, means were compared. A *p*-value of less than 0.05 was regarded to be of statistical significance.

## 3 Results and discussion

### 3.1 Method validation

With a relative standard deviation (RSDs %) of less than 3.3 %, the mean recovery of 3-MCPD and 1,3-DCP were 95.4–99.4 % and 94.1–99.3 %, respectively (Table 1). Based on the calibration plot, coefficients of determination (R^2^) for 1,3-DCP and 3-MCPD were 97.0–98.0 %. Also, the calculated LOD and LOQ were respectively 0.001 and 0.003 ng/mg (Table 1).

**Table 1:** Recoveries (%), relative standard deviation (RSD, %), and correlation coefficients (R^2^) obtained for 3-MCPD and 1,3-DCP (mg/kg) level determination.

Sample	Spiked concentration (mg/kg)		RSD%	R^2^	Spiked concentration (mg/kg)		RSD%	R^2^
	3-MCPD				1,3-DCP			
	0.5	1.0			0.5	1.0		
Low-fat mayonnaise	95.4	98.5	(2.5)	0.970	94.1	99.1	(3.3)	0.970
High-fat mayonnaise	96.5	99.4	(2.2)	0.975	96.2	99.3	(2.5)	0.980

### 3.2 Mean levels of 3-MCPD and 1,3-DCP

As shown in Table 2, the mean concentration of 1,3-DCP and 3-MCPD significantly differed between low-fat and high-fat samples as high-fat samples had significantly higher mean levels of 1,3-DCP and 3-MCPD than low-fat ones; however, no significant difference among the six brands was observed in this regard (Table 2).

**Table 2:** Mean concentration of 3-MCPD and 1,3-DCP in mayonnaise samples from six different brands.

Brand	Concentration of 3-MCPD (mg/kg)		Concentration of 1,3-DCP (mg/kg)	
	Low-fat mayonnaise	High-fat mayonnaise	Low-fat mayonnaise	High-fat mayonnaise
Brand A	0.030 ± 0.003^b^	0.050 ± 0.005^b^	0.008 ± 0.002^a^	0.020 ± 0.003^a^
Brand B	0.040 ± 0.005^ab^	0.080 ± 0.007^ab^	0.005 ± 0.002^ab^	0.010 ± 0.003^ab^
Brand C	0.020 ± 0.003^b^	0.040 ± 0.005^b^	0.005 ± 0.002^ab^	0.010 ± 0.003^ab^
Brand D	0.030 ± 0.003^b^	0.100 ± 0.050^a^	0.006 ± 0.002^a^	0.020 ± 0.003^a^
Brand E	0.050 ± 0.005^a^	0.070 ± 0.006^ab^	<LOD	0.008 ± 0.002^ab^
Brand F	0.060 ± 0.005^a^	0.100 ± 0.050^a^	<LOD	0.006 ± 0.002^ab^

LOD: Limit of Detection (0.001 ng/mg)

In each column, lowercase superscripts (a, b, etc.) express statistical variations among different brands. The values with at least one similar superscript are not significantly different from each other but those with different superscripts are significantly different.

It has been shown that 3-MCPD level is highly associated with the applied temperature as well as the amount of lipids and glycerol. High concentrations of 3-MCPD in some food products may be explained by the high temperature applied during production [5]. The highest concentration of esterified 3-MCPD found in food products was initially reported in dark malt (0.58 mg/kg) and French fries (6.1 mg/kg) and all refined oil and product samples with a lipid fraction containing refined fats were generally found to contain 3-MCPD [28]. The presence of free form of 3-MCPD in food is dependent on glycerin, temperature, lipid content, salt-containing molecules, and water content, however, the role of high temperature has been particularly highlighted [7], [8]. High temperatures may cause intricate chemical reactions in oil, leading to the denaturation of proteins and gelatinization of starches, all of which contributing to the distinct qualities of fried foods [1]. Also, effects of tert-butylhydroquinone, NaCl, and long-term deep-frying at 177 °C on the content of esterified 3-MCPD over a four-day period were examined and it was revealed that when the amount of NaCl in a fried starchy preparation consumed traditionally in China, decreased from 1.50 g/100 g to zero, esterified 3-MCPD level in the extracted oils decreased from 1.272 to 0.360 mg/kg [17]. Furthermore, Wong et al., assessed the influence of temperature (160 and 180 °C), frying time (100 min), and NaCl concentration (0, 1, 3, and 5 %) on the occurrence of esterified 3-MCPD in refrigerated, bleached, and deodorized palm olein during deep-fat frying of potato chips for five successive days and found that the ester level decreased by increasing the frying time, and that this trend became stronger at higher frying temperatures and greater concentrations of NaCl. Thus, it can be deduced that esterified 3-MCPD are unstable when heated for extended periods of time because decomposition rate was higher than the formation rate [40]. Thus, where a strong chloride ion donor capable of forming 3-MCPD in the oil exists, heat treatment increases the levels of this chemical [20]. When chloride ions are present, bidirectional conversion between 3-MCPD and glycidol or between their esterified forms can also occur [27]. Martin et al. indicated that high-temperature heating in the presence of HCl is what produces 3-MCPD in food products when sources like lecithin, glycerin, and glycerides are present [24]. Nevertheless, despite considerable research efforts made, detailed mechanism(s) of formation of these compounds remains unclear. The maximum level of 3-MCPD was found in certain samples of commercially refined oil. However, heat pretreatment of fruits/seeds such as roasting, may be one of the reasons of the raised 3-MCPD level in non-refined oils [29]. The main ingredients of mayonnaise are vegetable oil, egg yolk, vinegar, water, and sodium chloride. Mayonnaise is an oil-in-water emulsion. Food with high fat content (e.g. butter, margarine, and mayonnaise) were shown to have considerable levels of thermal processing contaminants [41]. Industrial processes involving edible oils have the potential to produce the toxic compounds 1,3-DCP and 3-MCPD [9]. During the refining process, triglycerides hydrolyze to form MCPD in vegetable oils. High temperatures and strong acids or bases can cause the triglyceride molecules to hydrolyze, thus releasing fatty acids and glycerol [26]. Production of low-fat mayonnaise is of importance to both consumers and the food industry. Replacers for fat may enhance processing capabilities and offer nutritional advantages. Guar, pectin, and xanthan gums are polysaccharide gels that work well as fat substitutes. To achieve the required rheological qualities, xanthan gum was added to mayonnaise either alone or in combination with other gums in salad dressings. Since citrus fiber has been used to replace fat and stabilize without negatively affecting the quality, it is a viable option for use in mayonnaise [3]. Due to the impracticality of conducting an exhaustive analysis of all food sources of such compounds, our investigation focused solely on the occurrence of these compounds in mayonnaise samples and oral exposure to them via mayonnaise consumption; thus, data presented in this study should be considered with caution. Besides, previous studies determined 3-MCPD content in its free form, ester form, or a combination of both [2]. The current investigation determined the levels of free form of the compounds in mayonnaise samples, and future studies should investigate esterified forms in mayonnaise.

### 3.3 Risk assessment

In the present probabilistic health risk assessment, the total HI values were below one which is interpreted as no major health risk (Table 3, Table 4). Based on the MCS model, at 50th, 80th, and 95th centiles, total HI values for 3-MCPD exposure were 3.92×10^−2^, 5.61×10^−2^, and 7.10×10^−2^, respectively (Tables 3) and for 1,3-DCP were 5.74×10^−3^, 8.22×10^−3^, and 1.03×10^−2^, respectively (Table 4).

**Table 3:** Probabilistic target hazard quotient (THQ) and hazard index (HI) values at the 50th, 80th, and 95th centile for oral exposure to 3-MCPD via consumption of mayonnaise samples.

Brand		Low-fat mayonnaise			High-fat mayonnaise	
	THQ (50th centile)	THQ (80th centile)	THQ (95th centile)	THQ (50th centile)	THQ (80th centile)	THQ (95th centile)
Brand A	1.75×10^−3^	2.51×10^−3^	3.18×10^−3^	2.92×10^−3^	4.20×10^−3^	5.30×10^−3^
Brand B	2.34×10^−3^	3.35×10^−3^	4.24×10^−3^	4.68×10^−3^	6.71×10^−3^	8.48×10^−3^
Brand C	1.17×10^−3^	1.67×10^−3^	2.12×10^−3^	2.34×10^−3^	3.35×10^−3^	4.24×10^−3^
Brand D	1.75×10^−3^	2.51×10^−3^	3.18×10^−3^	5.85×10^−3^	8.38×10^−3^	1.06×10^−2^
Brand E	2.92×10^−3^	4.20×10^−3^	5.30×10^−3^	4.10×10^−3^	5.87×10^−3^	7.42×10^−3^
Brand F	3.51×10^−3^	5.03×10^−3^	6.36×10^−3^	5.85×10^−3^	8.38×10^−3^	1.06×10^−2^
HI	1.34×10^−2^	1.92×10^−2^	2.44×10^−2^	2.57×10^−2^	3.70×10^−2^	4.66×10^−2^
HI (SUM) for low- and high-fat samples						
50th centile = 3.92×10^−2^						
80th centile = 5.61×10^−2^						
95th centile = 7.10×10^−2^						

THQ: Target Hazard Quotient

HI: Hazard Index

**Table 4:** Probabilistic target hazard quotient (THQ) and hazard index (HI) values at the 50th, 80th, and 95th centile for oral exposure to 1,3-DCP via consumption of mayonnaise samples.

Brand		Low-fat mayonnaise			High-fat mayonnaise	
	THQ (50th centile)	THQ (80th centile)	THQ (95th centile)	THQ (50th centile)	THQ (80th centile)	THQ (95th centile)
Brand A	4.70×10^−4^	6.71×10^−4^	8.50×10^−4^	1.17×10^−3^	1.67×10^−3^	2.12×10^−3^
Brand B	2.93×10^−4^	4.20×10^−4^	5.31×10^−4^	5.86×10^−4^	8.40×10^−4^	1.06×10^−3^
Brand C	2.93×10^−4^	4.20×10^−4^	5.31×10^−4^	5.86×10^−4^	8.40×10^−4^	1.06×10^−3^
Brand D	3.51×10^−4^	5.03×10^−4^	6.37×10^−4^	1.17×10^−3^	1.67×10^−3^	2.12×10^−3^
Brand E	NA	NA	NA	4.70×10^−4^	6.71×10^−4^	8.50×10^−4^
Brand F	NA	NA	NA	3.51×10^−4^	5.03×10^−4^	6.37×10^−4^
HI	1.40×10^−3^	2.01×10^−3^	2.54×10^−3^	4.33×10^−3^	6.20×10^−3^	7.85×10^−3^
HI (SUM) for low and high fat samples						
50th centile = 5.74×10^−3^						
80th centile = 8.22×10^−3^						
95th centile = 1.03×10^−2^						

THQ: Target Hazard Quotient

HI: Hazard Index

NA: not available

The highest MOE values were estimated for low- fat samples and the lowest values were for high-fat ones (Table 5). Based on the probabilistic approach, the average estimated MOEs were 5.17×10^4^, 6.95×10^4^, and 7.31×10^4^ respectively at the 50th, 80th, and 95th centiles for low-fat samples, (Table 5); therefore, it might be concluded that the exposure to chemicals via intake of the analyzed low- and high-fat mayonnaise samples was of *de minimis* public health concern at these centiles (Table 5).

**Table 5:** Probabilistic Margin of Exposure (MOE) values at the 50th, 80th, and 95th centile for oral exposure to 3-MCPD via consumption of mayonnaise samples.

Brand		Low-fat			High-fat	
	MOE (50th centile)	MOE (80th centile)	MOE (95th centile)	MOE (50th centile)	MOE (80th centile)	MOE (95th centile)
Brand A	5.80×10^4^	7.80×10^4^	8.20×10^4^	3.48×10^4^	4.67×10^4^	4.92×10^4^
Brand B	4.35×10^4^	5.84×10^4^	6.15×10^4^	2.17×10^4^	2.92×10^4^	3.07×10^4^
Brand C	8.70×10^4^	1.16×10^5^	1.23×10^5^	4.35×10^4^	5.84×10^4^	6.15×10^4^
Brand D	5.80×10^4^	7.80×10^4^	8.20×10^4^	1.74×10^4^	2.33×10^4^	2.46×10^4^
Brand E	3.48×10^4^	4.67×10^4^	4.92×10^4^	2.48×10^4^	3.34×10^4^	3.51×10^4^
Brand F	2.90×10^4^	3.90×10^4^	4.10×10^4^	1.74×10^4^	2.33×10^4^	2.46×10^4^
MOE (Average)	**5.17×10**^**4**^	**6.95×10**^**4**^	**7.31×10**^**4**^	**2.66×10**^**4**^	**3.57×10**^**4**^	**3.76×10**^**4**^

We found no significant difference among the six brands in the level of chloropropanols but a remarkable difference in this concern was observed between the two types of mayonnaise (low- and high-fat) samples and the level of these compounds were significantly higher in high-fat samples. The total HI values calculated for oral exposure to 1,3-DCP and 3-MCPD through intake of the analyzed mayonnaise samples were below one in the probabilistic assessment, indicating no health risk to consumers. In the probabilistic methodology concerning the MOE scenario, the potential exposure to chemical substances through the consumption of the examined low- and high-fat mayonnaise samples was deemed to be of *de minimis* public health significance at the specified centiles. In this study, we only determined the levels of the free form of 3-MCPD along with 1,3-DCP; in future studies, it is recommended to determine the levels of bound 3-MCPD in mayonnaise samples and make comparisons between the level of free and esterified compounds in this product.

## 4 Conclusion

In this work, we assessed the health risk associated with oral exposure to 3-MCPD and 1,3-DCP via consumption of two types of mayonnaise (low- and high-fat) by Iranian consumers. There was no significant difference among the six brands in the level of the examined chloropropanols. However, there was a statistically significant difference in the mean concentration of 3-MCPD and 1,3-DCP between the two types of mayonnaise samples and the level of these compounds were significantly higher in high-fat samples compared to low-fat ones. The total HI values calculated for oral exposure to 3-MCPD and 1,3-DCP were below one based on the probabilistic assessment, indicating no health risk to consumers. In the MOE, consumption of low- and high-fat mayonnaise samples was of *de minimis* health concern for oral exposure to 3-MCPD and 1,3-DCP, at the 50th, 80th, and 95th centiles. In future studies, the potential effect of the time period between production and analysis as well as the storage conditions should be examined. More frequent assessments on larger number of brands/samples are necessary to provide a clearer picture of occurrence of chloropropanols and potential oral exposure to these chemicals.

## CRediT authorship contribution statement

**Mahin Velayati:** Methodology. **Ghazaleh Tabriznia Tabrizi:** Formal analysis. **Ramin Rezaee:** Writing – review & editing, Supervision, Project administration, Funding acquisition. **Gholamreza Karimi:** Investigation, Conceptualization. **Seyedeh Faezeh Taghizadeh:** Writing – original draft, Software, Formal analysis. **Aristidis Tsatsakis:** Validation, Methodology, Conceptualization. **Christina Tsitsimpikou:** Writing – review & editing. **Hadi Haghparast:** Formal analysis.

## Declaration of Competing Interest

The authors declare that they have no known competing financial interests or personal relationships that could have appeared to influence the work reported in this paper.

## Data Availability

Data will be made available on request.
